# Books and Drug Men

**Published:** 1878-02-20

**Authors:** 


### BOOKS AND DRUG MEN.

"I am pleased with the practical character of your journal. Your advertising department is also useful and interesting to many practitioners who read little else besides The Record. To get what I want, I have only to see our druggist here, and have him order the articles from the advertising houses. Can I get a good work on midwifery in your city? Your review department is not as full as we would like. Would like to know the new books that come out, and have your opinion as to their merits," etc.

We extract the above from a letter received, because it verifies what we have often said in regard to the necessity and usefulness of an advertising department in a medical journal, and should furnish a valuable hint to advertisers, especially to druggists and book merchants. We have to confess that our book notices have been somewhat meagre, and we are sorry for it, but if authors of new books, and those who vend them, do not appreciate the importance of these notices in bringing their works before the profession, we can not help it. We notice, however, that certain Northern journals are liberally supplied with these new works for notice, under the impression, we suppose, that physicians in the South, or the readers of Southern journals, do not purchase books. This is a mistake. We have many inquiries like the above, and it would well repay the authors of new works to send them to us for review.

